# Older adults are mobile too!Identifying the barriers and facilitators to older adults’ use of mHealth for pain management

**DOI:** 10.1186/1471-2318-13-43

**Published:** 2013-05-06

**Authors:** Samantha J Parker, Sonal Jessel, Joshua E Richardson, M Cary Reid

**Affiliations:** 1 Department of Medicine, Weill Cornell Medical College, New York, NY 10065, USA; 2 The Center for Healthcare Informatics and Policy, Weill Cornell Medical College, New York, NY 10065, USA

## Abstract

**Background:**

Mobile health (mHealth) is a rapidly emerging field with the potential to assist older adults in the management of chronic pain (CP) through enhanced communication with providers, monitoring treatment-related side effects and pain levels, and increased access to pain care resources. Little is currently known, however, about older adults’ attitudes and perceptions of mHealth or perceived barriers and facilitators to using mHealth tools to improve pain management.

**Methods:**

We conducted six focus groups comprised of 41 diverse older adults (≥60 years of age) with CP. Participants were recruited from one primary care practice and two multiservice senior community day-visit centers located in New York City that serve older adults in their surrounding neighborhoods. Focus group discussions were recorded and transcribed, and transcriptions were analyzed using direct content analysis to identify and quantify themes.

**Results:**

Focus group discussions generated 38 individual themes pertaining to the use of mHealth to help manage pain and pain medications. Participants had low prior use of mHealth (5% of participants), but the vast majority (85%) were highly willing to try the devices. Participants reported that mHealth devices might help them reach their healthcare provider more expeditiously (27%), as well as help to monitor for falls and other adverse events in the home (15%). Barriers to device use included concerns about the cost (42%) and a lack of familiarity with the technology (32%). Facilitators to device use included training prior to device use (61%) and tailoring devices to the functional needs of older adults (34%).

**Conclusions:**

This study suggests that older adults with CP are interested and willing to use mHealth to assist in the management of pain. Participants in our study reported important barriers that medical professionals, researchers, and mHealth developers should address to help facilitate the development and evaluation of age-appropriate, and function-appropriate, mHealth devices for older persons with CP.

## Background

Chronic pain (CP) is a debilitating disorder that affects up to half of all adults age 65 and above in the United States [1]. The burden of CP in the United States is projected to increase with the aging of the U.S. population. Commonly occurring pain disorders that affect older adults include back pain, osteoarthritis, rheumatoid arthritis, and various neuropathies. The pain associated with these and other pain-producing conditions limits physical function [2,3], decreases quality of life [4,5], lowers perceived health status [3,6], and increases the risk for falls [7], as well as suicide [8]. Age is an important risk factor for both underassessment and undertreatment of pain [4].

Surveys of older adults with diverse pain problems indicate that they frequently use analgesic medications to treat pain [9,10]. However, commonly prescribed analgesics, including non-steroidal anti-inflammatory agents and opioid medications have significant side effects that limit use, particularly among older adults [11,12]. The side-effect profiles associated with these medications make many older adults reluctant to use them thereby limiting effectiveness [13]. In addition, the long-term safety and efficacy of these medications remain to be defined [14]. There is a pressing need to improve CP management strategies given established risks associated with many pharmacotherapies [2,3,11,12], and uncertainty regarding their long-term effects [14].

Mobile health technologies (mHealth) have the potential to improve care delivery to older adults with CP. mHealth represents a subset of telehealth [15] that leverages mobile information-communication technology devices such as smartphones and tablets, as well as software applications for each that can provide portable and patient-level healthcare support remotely. mHealth could potentially track daily pain levels, monitor symptom levels (e.g., fatigue, depressive symptoms), capture treatment-related side effects, remind patients to take analgesic medications, provide educational and self-management materials to patients, facilitate timely interactions with health care providers, and provide social support to older adults who experience pain [16]. Additionally, these devices could be used as in-home or wearable monitors that could track patient activities of daily living. For example, they could monitor an individual’s level of physical activity or alert caregivers if an individual experiences a fall or near fall [17].

Research suggests that non-mobile telehealth interventions can broadly support the treatment of CP among younger [18-20] and older [21,22] adults. However, little research [17] has focused on mobile technology’s effects (or potential effects) on healthy aging despite it being listed as a key intervention by The Centers for Disease Control and Prevention [23]. In sum, it remains unclear whether and to what extent older adults will adopt mHealth to help manage their pain and/or pain treatments.

We therefore sought to examine the willingness of older adults with CP to adopt mHealth technologies, and to identify participants’ perceived barriers and facilitators to adopting mHealth technologies. Understanding older adults’ perceived barriers and facilitators to mHealth could inform device design and consumer adoption strategies to improve pain care in this rapidly expanding patient population.

## Methods

### Design

This qualitative study employed a focus group approach with semi-structured questions to generate primary data.

### Study sites

Focus groups were conducted in three sites in New York City: 1) New York-Presbyterian’s (NYP) Wright Center on Aging, 2) Lenox Hill Senior Center, and 3) Central Harlem Senior Center. The Wright Center on Aging is Cornell/NYP’s Division of Geriatrics’ outpatient practice and mainly serves non-Hispanic white older adults. Lenox Hill Senior Center and Central Harlem Senior Center serve older (60 years and above) adults who are mostly non-Hispanic white (at Lenox Hill) or African American (at Central Harlem). Services provided at the two senior centers include daytime socialization, educational and health promotion activities, and daily meals. The three sites were chosen to provide a diverse group of older participants from clinical as well as non-clinical sites. All three facilities serve community-dwelling older adults living independently in New York City, except that about 10% of patients served at the Wright Center on Aging reside in assisted-living facilities. We conducted six focus groups in total (two per site), and focus groups at each site were comprised only of participants recruited from that site. The Weill Cornell Institutional Review Board approved this study.

### Participant recruitment

We carried out two different recruiting strategies in the outpatient practice and the senior centers to identify and gain commitment from prospective participants. First, we recruited a convenience sample of patients from the Wright Center on Aging outpatient practice with the assistance of their primary care physicians. All Wright Center attending physicians (N = 12) were informed about the study in a group practice meeting. The physicians were presented with a list of their patients (those seen in the past year) and were asked to identify potentially eligible participants. Five practice physicians agreed to contact prospective participants by phone to explain the study and determine whether the patients identified after review of their patient rosters (N = 13) would be willing to speak to the research team about participating in the project. Patients were eligible to participate in the study if they were: 1) 60 years of age or older; 2) had a self-reported CP problem greater than 3 months due to a condition other than cancer; and 3) judged by their physician to be likely to participate fully in a focus group discussion. All prospective participants contacted by the physicians gave approval to be contacted by a member of the research team. Of the 13 patients contacted by telephone, 11 agreed to participate. The remaining two patients had prior engagements during the times focus groups were scheduled.

Second, to recruit prospective participants at the two senior centers, one member of the research team (SJP) explained the study during routine lunchtime announcements at each center and recruited interested participants. Senior center lunchtime attendance (counted in number of lunch meals served daily) was 125 adults at both sites during participant recruitment. Of the 125 adults visiting the two centers during lunchtime announcements, 40 (32%) individuals expressed interest. One investigator (SJP) recorded all names and phone numbers of interested senior center clients and phoned them on an as-needed basis until a sufficient number was obtained to populate each focus group. All senior center members were eligible to participate if they reported experiencing pain for three or more months at the time of the telephone call and spoke English. Interested participants were recruited based upon their availability to attend one of the focus groups. Each participant selected a time that was convenient from the groups offered. There was no attempt to ensure homogeneity of participants (e.g., in terms of gender, age, pain intensity) across the groups. Thirty of the 40 individuals participated in a focus group (about one in 4 persons who were present at the lunchtime announcements). Of the 10 older adults who did not participate, all cited scheduling conflicts as a reason for not participating. Participants did not differ from non-participants with respect to age, gender or race/ethnicity status (data not shown).

### Focus group methodology

The research team reviewed published mHealth-related literature [17-22] to generate an interview guide with semi-structured questions and follow-up probes. We specifically reviewed the literature for 1) types of mHealth available, 2) proposed use of mHealth in aging populations and 3) use of mHealth for pain management. The interview guide was pilot tested with Wright Center patients prior to its use with study participants.

During the focus groups, one investigator (SJP) introduced the study, explained the focus group objectives and process, introduced mHealth technologies, displayed a smartphone (an iPhone 4), and moderated the discussions. One other team member (SJ) took notes during the sessions. Participants were asked to share any prior experiences using mHealth in healthcare situations, discuss whether having a mHealth device would make them more comfortable when starting a medication (e.g., acetaminophen, NSAID, opioid) to treat their pain (“Would this technology make you feel more comfortable taking analgesic medications for your pain?”), describe their willingness to use mHealth as a tool to better manage their pain or pain treatments (“How willing would you be to try one or more of these technologies in caring for your chronic pain or pain treatment?”) and describe barriers (“What barriers do you see in using this technology?”), as well as facilitators to using mHealth devices around their pain disorder (“What facilitators do you see in using this technology?”). [See Appendix for the full focus group guide.]

At the end of each focus group, all participants completed a 14-item self-administered questionnaire that inquired about participants’ demographics (e.g., age, gender, race/ethnicity status), pain intensity (on a 0-to-10 scale), interference with daily activities due to pain (Brief Pain Inventory Short Form) [24], and the number of days of inactivity in the past month that occurred as a consequence of pain [25]. The questionnaire also asked participants if they used (for whatever reason) any of the following: personal computers, the Internet, cell phones, smartphones [iPhone, Android, etc.], computer tablets, personal digital assistants, or any other communication technology devices.

### Analysis

All focus groups were audio recorded, transcribed, and analyzed via directed content analysis [26]. [SJP and SJ analyzed the qualitative data after each focus group.] The transcripts were read and then preliminary codes were generated around user needs and potential barriers and facilitators. The basic unit of analysis consisted of discrete sections of text that the investigators agreed conveyed a complete idea or construct. For example the phrase, “The size of the equipment… is too small. I want something … larger, bigger something I can see,” was coded as, “make equipment more user friendly for older adults with functional limitations.” In an iterative process, two investigators (SJP, SJ) independently analyzed the focus group data, routinely met to discuss their observations, reconciled divergent interpretations to agree on a common set of themes, and then conducted additional focus groups to further inform the theme development process. Three investigators (SJP, SJ and MCR) re-reviewed the transcripts, coded the text by theme, and then counted the number of instances each theme appeared. Focus group sessions continued until the investigators reached thematic saturation, i.e., researchers agreed that the data generated from the previous two focus groups produced no new themes [27].

## Results

A total of 41 subjects participated in 6 focus groups (2 at each study site) during July 2012. Focus groups ranged in size from 5 to 9 participants, and lasted between 43 and 65 minutes. The average age of participants was 76.2 years, 56% identified as non-Hispanic white, while 34% identified as African American (Table 1). Participants were mostly female (78%) and reported an average pain intensity level of 5.0 (0-to-10 scale) over the 2 week period prior to the focus group. Participants reported varying use of information-communication technologies: 59% used cell phones, 41% employed personal computers, and 41% regularly searched the Internet, while 17% used tablet devices and 7% reported owning a smartphone. Participants did vary by site by race/ethnicity. Wright Center on Aging and Lenox Hill Senior Center participants were predominantly non-Hispanic white (85%), while most Central Harlem Senior Center participants identified as African-American (92%).

**Table 1:** Characteristics of study sample N = 41


Age, mean ± sd	76.2 ±9.3
Race/ethnicity status	
Non-Hispanic White, n (%)	23 (56.0)
African-American, n (%)	14 (34.0)
Other, n (%)	4 (10.0)
Female, n (%)	32 (78.0)
College level education or higher, n (%)	23 (56.0)
Average pain intensity over past 2 weeks (0–10), mean ± sd	5.0 ±2.6
Pain duration in years, mean ± sd	7.2 ±13.2
Type of pain disorder	
Degenerative joint disease, n (%)	21 (51.0)
Low back pain, n (%)	17 (41.0)
Other^*^	24 (59.0)
Uses medication to manage pain, n (%)	22 (54.0)
Uses physical therapy to manage pain, n (%)	16 (39.0)
Uses other pain management techniques^**^	22 (54.0)
Days of inactivity due to pain in the past month, mean ± sd	4.8 ±6.6
Current use of technology	
Cell phone, n (%)	24 (59.0)
Personal computer, n (%)	17 (41.0)
Internet, n (%)	17 (41.0)
Tablet device, n (%)	7 (17.0)
Smartphone, n (%)	3 (7.0)

^*^ Participants could cite more than one kind of pain problem; other causes of pain included injury, Takayasu arteritis, and orofacial pain.

^**^Other techniques included interventional therapies, psychological therapies, and alternative medicines.

### Summary of focus group results

Focus group discussions generated 38 individual themes pertaining to the use of mHealth to help manage pain and pain medications (Table 2). Of the 38 themes, 8 involved participants’ potential use of (or willingness to use) mHealth devices; 8 described potential pain care improvements participants felt could occur as a consequence of employing mHealth technologies (e.g., help reach a healthcare provider); 14 described perceived barriers to using mHealth, and 8 described facilitators that could increase the likelihood that older adults would use the devices. Two participants (5%) reported using an mHealth-specific technology. One participant used calendar reminders on a smartphone to manage daily medications and the other accessed health-related websites via a mobile tablet.

**Table 2:** Selected themes reported by participants^*^

	**N (%) reporting each theme**
**Concerns about mHealth use**	
Reluctance to rely on a machine	8 (19.5)
Feel like they “don’t need it”	7 (17.3)
Concerned about whether healthcare provider will receive information generated by device	6 (14.6)
**Ways mHealth devices might be used**	
Help reach healthcare provider more expeditiously	11 (26.8)
Monitor over 24-hour period	10 (24.4)
Monitor for falls and other adverse events in the home	6 (14.6)
Provide 2 way communication channel between physician and patient	6 (14.6)
Facilitate sharing of information (with physician/family members)	3 (7.3)
Provide supervision and sense of security	4 (9.8)
Facilitate evaluation of treatment outcomes	1 (2.4)
**Barriers to mHealth use**	
Concern about battery dying	20 (48.8)
Cost	17 (41.5)
Lack of familiarity with technology	13 (31.7)
Forgetfulness/memory problems	12 (29.3)
Concerns about privacy	8 (19.5)
Unwilling to wear monitor	4 (9.8)
Concern about functional limitations	4 (9.8)
Concern about learning to use technology	3 (7.3)
Concern about device malfunction/incorrect use by patient	3 (7.3)
Health problems too complex	1 (2.4)
Concern about lack of human interaction	1 (2.4)
No primary care physician	1 (2.4)
Technology connection problems in apartment building	1 (2.4)
**Facilitators to mHealth use**	
Provide training on device use	25 (61.1)
Tailor equipment to older adults’ functional abilities	14 (34.1)
Employ information technology support staff	10 (24.4)
Evidence that mHealth device use leads to improved pain outcomes	2 (4.9)
Wearable mHealth monitors (as opposed to use of wall/home mounted monitors)	2 (4.9)

^*^A total of 38 themes were identified. Other themes included 1) time frame for response to health crises, 2) suggestion that the device should also function as a phone (be multi-functional), and 3) concern about the expertise of the provider monitoring the device.

#### Willingness to use mHealth

The vast majority (85%) of participants reported that they were very willing to use mHealth to help manage their pain condition:

“I would be willing to use something like that, I’ve never used anything, but I’m definitely willing to try it… a lot of times when you’re in pain…and have to call a physician…it takes…a while to get to the telephone to call someone.”

Seven participants (17%) said that they did not need mHealth to help monitor pain-related problems or symptoms, while 15% expressed concerns about whether the information generated from the device(s) would reach their provider in a timely manner. Participants stated they would use mHealth technologies if the devices were connected to a doctor/healthcare provider, or facilitated communication between patients and healthcare providers or emergency services. Participants expressed that connectivity could increase older adults’ comfort with technology in general, and help doctors to assess pain problems in the home environment:

“I would see [using mHealth] only if it was connected to the doctor…I think I have a pretty good idea about how I feel every day. But perhaps doing it where the doctor could evaluate it…you could have this chart with what happened [showing pain levels] over 6 months. Maybe [the physician] would see something.”

Participants noted several potential ways mHealth could help to improve pain care, including assisting patients to reach healthcare providers more expeditiously (27%), provide 24-hour monitoring (24%) to include monitoring for falls and other adverse events at home (15%):

“Well I’ve fallen once, so I would love to have something that could advise me that I’m going to fall… whether I’m taking medication or not…”

Another participant noted:

“I could have used something like that, because I was falling a lot over a period of several years. This [mHealth device] could’ve have been helpful because I really never knew when I was going to fall, and it seemed like my condition was escaping a diagnosis, until my physician realized I was taking too much medication. I stopped the medication and stopped falling. So this [device] would’ve been very, very helpful.”

#### Barriers to using mHealth

Participants described multiple potential barriers to using mHealth (Table 2). Cost was a concern noted by 41% of participants (while 12% declared cost was not a barrier). The latter group felt the cost was worth the comfort of having an mHealth technology available:

“I just came out of the hospital Saturday, and I think if you need that phone, go get it…$30 a month more or less [for a data plan], because it’s a way of knowing if you’re sick or not sick.”

Some participants (19%) were concerned with an invasion of privacy from mHealth tracking their movements. Conversely, 12% of participants were not concerned about privacy and felt that tracking their movements would make them feel less isolated while managing their pain condition:

“I wouldn’t mind because I’m alone, and I would feel a sense of security if I knew someone was watching.”

Functional limitations were expressed by about one in three participants (Table 2). These limitations ranged from visual and auditory deficits, to forgetfulness and concerns about their ability to keep track of a device. Many of the older adults felt that it would be important to make mHealth technologies that address these limitations:

“…I have macular degeneration and glaucoma, and I am eventually not going to be able to read or see. So [mHealth usability] would have to be considered somewhere down the road.”

#### Facilitators to using mHealth

Focus group participants were enthusiastic about the potential use of mHealth technology to assist in pain management. A majority of participants reported that mHealth training that targets the needs of older adults would be necessary and useful:

“Train us! That’s what we need!”

Another said:

“It seems like if you have a training facility you could reach more individuals that might be able to use the device and therefore you could program it to those people.”

Similarly, participants were interested in having the technology tailored and simplified to allow use by older adults with functional limitations such as those with poor eyesight or diminished dexterity (34%). As one participant stated:

“It has to be something that’s really easy to use. Simplified, very simplified. Maybe something like just pressing a button…like pressing one, two, or three. And one would mean something and two would mean something else.”

And:

“…it would be wonderful if [the mHealth device] was large enough where the actual numbers or words could [allow you to] read the doggone thing.”

Finally, no differences were seen between the themes discussed or frequency with which the themes were discussed between the African American and non-Hispanic white focus groups. Additionally, no differences were seen in responses based on participant age.

## Discussion

Our study suggests that older adults with self-reported CP are willing to use mHealth technologies and express a strong desire to learn how mHealth technologies can improve pain care. To date, research has demonstrated the accessibility, simplicity, and reliability and efficacy of mobile devices for pain care for non-elderly adults that reportedly facilitate more effective pain monitoring [28-31]. Few studies, however, have focused on older adults. To our knowledge, our study is the first to investigate mHealth specifically directed at pain management by ascertaining older adults’ opinions, as well as their perceived barriers and facilitators regarding mHealth device use.

Our findings contradict the stereotype that older adults are unwilling to try new technologies [32]. This misconception and others may contribute to a “technologic divide” that separates older adults from younger-aged adults who use these technologies routinely [33]. Paradoxically, however, smartphone use in certain segments of older adults is growing at a faster rate than use among other age groups [34]. These trends highlight the need to better understand the needs of older adults and the ways in which mHealth may address those needs.

Our data show that while most older adults with CP lacked prior exposure to mHealth and voiced some concerns about their lack of familiarity with the devices, most were very willing to use mHealth in an effort to improve their pain care. This finding is consistent with past research which documented older adults’ willingness to use banking ATM machines [35]. We found factors deemed most important for mHealth adoption by older patients included affordability and training in device use. In addition, bi-directionality was noted as an important feature of potential mHealth pain management devices. Participants thought bi-directionality could help address limitations in current healthcare, including increased access to healthcare providers and emergency services. These tools could be particularly helpful to older adults who may be isolated (e.g., live alone) or have limited access to pain care.

Training sessions and support for older adults learning technology could have a large impact on increasing the number of older adults using these technologies for pain management. Training older adults how to use mHealth devices could take place in a variety of healthcare settings including outpatient clinics, visiting home services or the inpatient setting. Additionally, trainings could be offered in community-based organizations that serve older adults such as senior centers, naturally occurring retirement communities (NORCs) and social adult day services. Community-based programs provide lifeline services to older adults and are part of the continuum of care in later life. Given this reach and infrastructure, such community-based programs could leverage current resources to support the dissemination of mHealth technologies. Doing so could both increase older adults’ exposure to mHealth and enhance their ability to access health information and self manage chronic conditions.

Our results indicate that older adults’ functional and cognitive limitations will likely necessitate technologies and programs customized to this user group. This result supports the need for research to inform mHealth device designs that specifically accommodate older adults with CP, as well as general efforts regarding mHealth for chronic disease management among older adults. Our study also supports research that seeks to optimize existing technologies (e.g., iPads) for use by older adults. While studies have begun to investigate optimizing technology for older adults [20], substantial work remains to be done. Prior research on mHealth that has included older adults has shown that older adults welcome mHealth interventions like home monitors [16], while their primary concerns were a potential invasion of privacy and whether they had sufficient ability to use the devices. Older adults in our study raised similar concerns about privacy, functional limitations and user-friendliness of technology. Our research confirms these concerns and provides suggestions for design features such as enlarged screens and enhanced volume control that could optimize use of mHealth devices by older adults. Our research adds an additional layer of complexity to privacy concerns, and suggests that older adults with more significant health problems, as well as those who live alone, may find the security provided by home monitors outweighs their concerns about privacy.

Our study has several limitations that warrant consideration. Our focus groups were convenience samples comprised of selected older patients (Wright Center on Aging) and self-selecting older adults (i.e., those attending two New York City senior centers) with pain problems. While all three sites serve many older adults, only a small number participated in the study. These aspects of our sample may limit the generalizability of our findings. In addition, while we did not record the total number of adults coming to the senior centers each day or those who would be eligible due to a CP problem, it is estimated that as many as 50% of community-dwelling older adults report experiencing a CP disorder [1]. Our focus groups included urban-dwelling older adults who may have expressed different needs compared to those living in suburban or rural areas. Additionally, while our sample did include both African American and non-Hispanic white adults, we did not include other cultural groups or non-English speaking participants. Similarly, participants in our study had a self-identified pain problem, and we made no attempt to control for average level of pain or years with pain. It is possible that a homogenous group of adults (with greater or fewer pain problems) would have expressed different opinions about the role of technology in pain care. Finally, while we did not find any differences in responses between older-old and younger-old participants in our focus groups, it is possible that homogeneous focus groups of older-old adults would have generated different responses and opinions.

## Conclusions

Our study indicates that older adults with CP are willing and interested in utilizing mHealth to assist in the management of their pain problems. The older adults who participated in our study reported important barriers that medical professionals, researchers, and mHealth developers can address to help facilitate the development and evaluation of age-appropriate and function-appropriate mHealth devices for use in older populations. Finally, older adults with CP will likely need targeted training and customized devices tailored to their needs and functional abilities. This paper offers a snapshot of older adults’ perceptions about pain management and provides a starting point for further investigation into the use of mHealth among older adults with CP.

## Appendix

Focus Group Questions

1. Mobile Health (mHealth) technologies are defined as information and communication technologies that deliver healthcare services remotely. mHealth includes portable devices, such as accelerometers and smartphones, and home devices, such as motion detectors.

a. Have you used mHealth in your medical care?

b. If you have used mHealth in your medical care, did you find it helpful?

2. Imagine a mobile health technology, such as a smartphone application, designed to help in the treatment of chronic pain. It could be a:

3. Symptom monitor: the technology would allow ongoing surveillance of both positive treatment effects (such as pain reduction) and negative treatment effects (such as sedation, loss of appetite, constipation)

4. Motion sensor: the technology would provide real-time data on activity levels as well as detect certain types of negative treatment events (falls, near-falls)

5. Bidirectional communication: the technology would allow real-time doctor-patient communication on the progress of treatment

a. How willing would you be to try this technology in caring for your chronic pain?

b. Would this technology make you feel more comfortable taking analgesic medications prescribed for your pain?

c. What barriers do you see in using this technology?

d. What could facilitate your use of this technology?

e. What other thoughts do you have on using mHealth in treatment of your chronic pain (and among other patients like you)?

## Competing interests

The authors declare that they have no competing interests.

## Authors’ contributions

SJP assisted with study design, had primary responsibility for collecting the data, and participated in data analysis and interpretation and drafting the manuscript. SJ assisted with data collection, analysis and interpretation and helped to draft the manuscript. JR assisted with data analysis and helped to draft the manuscript. MCR designed the study, assisted with data analysis and interpretation and helped to draft as well as revise the manuscript. All authors read and approved the final manuscript.

## Pre-publication history

The pre-publication history for this paper can be accessed here:

http://www.biomedcentral.com/1471-2318/13/43/prepub
